# Direct Visualization of Chemical Cues and Cellular Phenotypes throughout Bacillus subtilis Biofilms

**DOI:** 10.1128/mSystems.01038-21

**Published:** 2021-11-23

**Authors:** Sarah M. Yannarell, Dusan Veličković, Christopher R. Anderton, Elizabeth A. Shank

**Affiliations:** a Department of Microbiology and Immunology, University of North Carolina at Chapel Hill, Chapel Hill, North Carolina, USA; b Environmental Molecular Sciences Laboratory, Pacific Northwest National Laboratory, Richland, Washington, USA; c Department of Systems Biology, University of Massachusetts Chan Medical School, Worcester, Massachusetts, USA; d Department of Microbiology and Physiological Systems, University of Massachusetts Chan Medical School, Worcester, Massachusetts, USA; University of California, Santa Cruz

**Keywords:** mass spectrometry imaging, MALDI-FTICR, confocal microscopy, multimodal imaging, metabolomics, gene expression, *Bacillus subtilis*, biofilms

## Abstract

Bacillus subtilis is a soil bacterium that can form biofilms, which are communities of cells encased by an extracellular matrix. In these complex communities, cells perform numerous metabolic processes and undergo differentiation into functionally distinct phenotypes as a survival strategy. Because biofilms are often studied in bulk, it remains unclear how metabolite production spatially correlates with B. subtilis phenotypes within biofilm structures. In many cases, we still do not know where these biological processes are occurring in the biofilm. Here, we developed a method to analyze the localization of molecules within sagittal thin sections of B. subtilis biofilms using high-resolution mass spectrometry imaging. We correlated the organization of specific molecules to the localization of well-studied B. subtilis phenotypic reporters determined by confocal laser scanning fluorescence microscopy within analogous biofilm thin sections. The correlations between these two data sets suggest the role of surfactin as a signal for extracellular matrix gene expression in the biofilm periphery and the role of bacillibactin as an iron-scavenging molecule. Taken together, this method will help us generate hypotheses to discover relationships between metabolites and phenotypic cell states in B. subtilis and other biofilm-forming bacteria.

**IMPORTANCE** Bacterial biofilms are complex and heterogeneous structures. Cells within biofilms carry out numerous metabolic processes in a nuanced and organized manner, details of which are still being discovered. Here, we used multimodal imaging to analyze B. subtilis biofilm processes at the metabolic and gene expression levels in biofilm sagittal thin sections. Often, imaging techniques analyze only the top of the surface of the biofilm and miss the multifaceted interactions that occur deep within the biofilm. Our analysis of the sagittal planes of B. subtilis biofilms revealed the distributions of metabolic processes throughout the depths of these structures and allowed us to draw correlations between metabolites and phenotypically important subpopulations of B. subtilis cells. This technique provides a platform to generate hypotheses about the role of specific molecules and their relationships to B. subtilis subpopulations of cells.

## INTRODUCTION

Across many different environments, microbes form complex communities embedded in self-produced extracellular matrices, known as biofilms (1). This extracellular matrix can serve as a protective mechanism in times of stress (2, 3). The resulting stratified biofilm structure gives rise to cellular differentiation or heterogeneous phenotypic and metabolic processes that are essential for cell survival (4–7). These differentiated processes can range from changes in primary metabolism and specialized (secondary) metabolite biosynthesis (8–11) to alterations in cellular phenotypes (such as the production of motile, extracellular-matrix-producing, or sporulating cells). Diverse tools are available for understanding the distribution of these metabolic and cellular processes within bacterial biofilms, including confocal laser scanning fluorescence microscopy, stable isotope labeling and Raman spectroscopy, transcriptomics and proteomics, and mass spectrometry imaging (12, 13).

Here, we developed a method to obtain accurate insights into how cellular gene expression programs correlate to the location of secreted metabolites within sagittal thin sections of Bacillus subtilis NCIB3610 colonies. B. subtilis is a soil-dwelling bacterium that forms robust colony biofilms on the agar-air interface when grown on a biofilm-inducing medium, MSgg (14–16). B. subtilis produces an exopolysaccharide and protein structural components that are critical to the surface adherence, stress tolerance, and architecture of its biofilms. These proteins are BslA, a hydrophobin encoded by *bslA*, TasA, an amyloid fiber that provides structural integrity to the biofilm, and TapA, an anchoring protein that attaches TasA to bacterial cell walls, both of which are encoded by the *tapA-sipW-tasA* operon (17–19). Because both TasA and TapA are essential for biofilm architecture and are highly upregulated during biofilm development, we used a *tapA* reporter as an indicator for biofilm formation (20). Previous work has shown that these biofilm matrix components are heterogeneously expressed across B. subtilis biofilms (20). In addition to cells expressing biofilm matrix components, other cell types have been described as being present within B. subtilis biofilms, including those that are motile (expressing the *hag* gene necessary for flagella) and those that are expressing specialized metabolites such as bacillibactin (*dhbA*), plipastatin (*ppsA*), subtilosin (*sboA*), surfactin (*srfAA*), and cannibal toxin production (*sdpA*) (21–23). Some of these specialized metabolites (e.g., surfactin and bacillibactin) have been implicated as having intraspecific signaling roles important to or associated with biofilm formation (24, 25). However, many of the relationships between bacterial metabolites and cellular behaviors (whether causal or simply correlated) remain unknown. Because B. subtilis biofilm formation and phenotypic heterogeneity are so well studied, we reasoned that it would be a useful bacterial model in which to correlate cellular gene expression patterns with specialized metabolites within biofilm structures to gain insights into phenotypic heterogeneity and bacterial metabolism.

**FIG 1:**
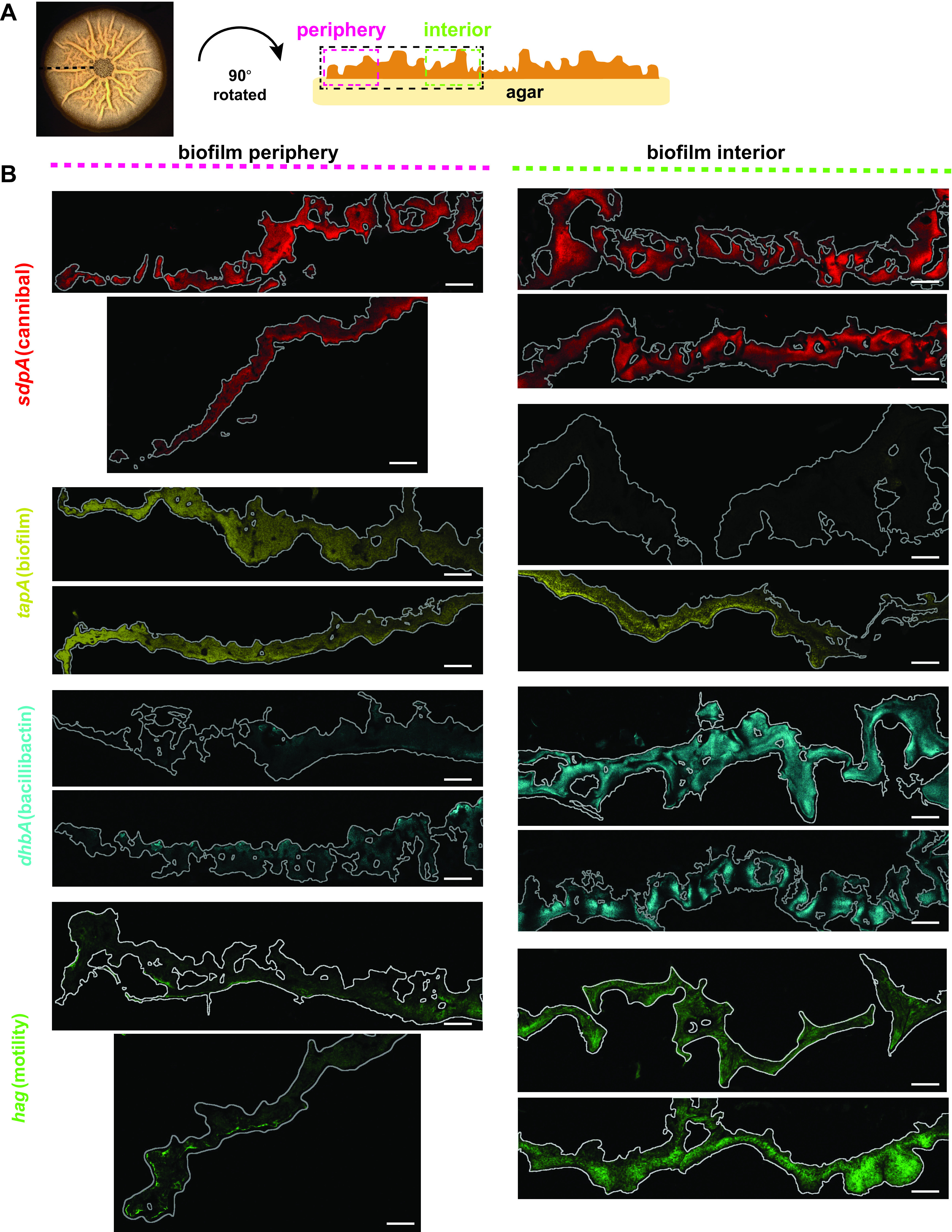
Confocal fluorescence images of thin-sectioned B. subtilis biofilms. (A) Schematic of a B. subtilis biofilm grown on MSgg medium and thin sectioned along the sagittal plane for microscopy and mass spectrometry analysis. (B) Using a 20× objective, we could visualize a single slice of thin-sectioned biofilms containing *YPet* reporters for genes encoding components of physiological or metabolite machinery at the interior and periphery. Colonies are outlined in gray. Brightness was linearly adjusted in the same way for each reporter using Fiji; therefore, intensity cannot be compared between reporters but can be compared across micrographs from the same reporter. Each reporter was false colored independently for ease of visualization. Bars, 100 μm.

Mass spectrometry imaging (MSI) is a powerful technique capable of obtaining chemical profiles directly from the surface of biological samples in an untargeted and unlabeled fashion (26, 27). Traditionally, to analyze the metabolome of bacterial colonies by MSI, colonies grown on agar are dried down and analyzed from the top surface (28). Matrix-assisted laser desorption/ionization (MALDI)-MSI on B. subtilis at the colony level has been used to identify and investigate molecules that mediate interspecies competition (29–33) and make progress toward understanding their impact on intraspecies communication (29, 34). However, this method averages the molecular signals over several vertical cell layers of the biofilm down into a single voxel. Given how much topography is present within B. subtilis colonies (Fig. 1A), this approach of MS imaging of the top surface will likely not fully capture the metabolic heterogeneity occurring between the top and bottom layers of the biofilm, where cells are known to differentiate into subpopulations with distinct phenotypes (20, 35, 36). Furthermore, imaging from the top does not provide information about the diffusion of metabolites into the agar medium below, the surface where the colony is grown. An alternative approach is to conduct MSI on cross-sectioned bacterial colonies. This method has been used to analyze interactions between B. subtilis and Streptomyces coelicolor, as well as Candida albicans and Pseudomonas aeruginosa in a three-dimensional (3D) fashion (37). Although this approach uniquely allows for the distributions of metabolites to be visualized deep within biofilm structures, it has seen surprisingly limited applications since it was first described, nor has it been paired with other imaging modalities.

Pairing MSI with microscopy techniques, for instance combining MSI with fluorescence *in situ* hybridization (FISH) (38) and metaFISH (a combination of high-resolution atmospheric-pressure MALDI-MSI and FISH) to visualize host-microbe interactions (39), has revealed details about microbial behavior that neither one of these techniques alone could reveal. We wanted to build on the established success of these multimodal approaches by combining, in parallel, MSI with confocal fluorescence microscopy on separate cross-sectioned bacterial colonies to determine how molecular cues correlate to cellular phenotypes at the community level within biofilms. In order to accomplish this, we first had to develop a parallel analysis pipeline for conducting MSI and confocal fluorescence microscopy in biofilms. Specifically, we needed to improve the existing sample handling and embedding methods for sectioning agar-based microbial colonies to preserve the stratified biofilm structure.

By gaining high-resolution spatial information about the heterogeneous distributions of cells and molecules throughout B. subtilis biofilms, we have identified molecules that have distinct, but previously uncharacterized, patterns of spatial localization, some of which correlate with cell-type-specific gene expression patterns. By visualizing the localization of molecules deep within biofilms, we can therefore generate testable hypotheses about the relationships between molecules and cellular transcriptional states, enabling us to interrogate their potential role as intraspecific cell-cell signals within B. subtilis biofilms.

## RESULTS AND DISCUSSION

### Heterogeneity in gene expression throughout B. subtilis biofilms visualized by confocal fluorescence microscopy.

To study B. subtilis gene expression within the 3D depth of the biofilm, we first constructed strains containing fluorescent transcriptional reporters for a subset of B. subtilis genes involved in encoding products important for motility (*hag*), extracellular matrix (*tapA*), and the specialized metabolites bacillibactin (*dhbA*) and the cannibal toxin (*sdpA*) (see Table S1 in the supplemental material). Some of these genes are well studied and are known to exhibit heterogeneous expression throughout the colony (21, 40–43), while others, including the specialized metabolite genes, have not yet been demonstrated to be spatially heterogeneously expressed throughout the colony. To construct these reporters, we introduced the fluorescent protein YPet (a variant of yellow fluorescent protein) under the control of promoters for these genes of interest and incorporated them into a neutral ectopic site on the B. subtilis genome (44).

10.1128/mSystems.01038-21.3 TABLE S1 Primers, plasmids, and strains used in this study. Download Table S1, PDF file, 0.1 MB. Copyright © 2021 Yannarell et al. 2021 Yannarell et al. https://creativecommons.org/licenses/by/4.0/ This content is distributed under the terms of the Creative Commons Attribution 4.0 International license.

Given the extensive heterogeneous gene expression occurring within B. subtilis biofilms, it was not initially clear that individual cross sections from different biofilms would reproducibly capture these phenomena. We therefore first needed to determine whether the 3D spatial distributions of gene expression of these cell type reporters within B. subtilis were consistent across biological replicate colonies. To do so, we grew biofilms of B. subtilis strains containing these different phenotypic fluorescent reporter constructs on MSgg for 48 h, vapor fixed the colonies (to prevent changes in fluorescence during processing), embedded the colonies in agarose, and thin sectioned the embedded biofilms for microscopy. A B. subtilis colony biofilm and a schematic of the resulting thin section are shown in Fig. 1A. We embedded the colonies with a top layer of agarose before sectioning to provide structure and avoid colony collapse during slicing. Previous B. subtilis thin sectioning work (20) used OCT (optimal cutting temperature) compound as an embedding agent, but OCT is incompatible with our downstream MSI. Although a single time point is admittedly limited at capturing the full picture of biofilm formation in B. subtilis, we chose a 48-h endpoint for these experiments to capture a time where at least some genes (including *tapA*) are known to be highly heterogeneously expressed in B. subtilis on MSgg (20). We then used confocal fluorescence microscopy to analyze biofilm thin sections from biological replicates of B. subtilis colonies to identify areas within the stratified colony where fluorescence (i.e., cell-type-specific gene expression) was observed.

We obtained replicate confocal fluorescence microscopy images from independently grown biofilms of strains containing fluorescent transcriptional reporters for cannibal toxin production (*sdpA*), extracellular-matrix production (*tapA*), bacillibactin (*dhbA*), and motility (*hag*); these replicates displayed similar expression levels and localization patterns in thin sections from replicate B. subtilis colonies (Fig. 1B). In thin sections visualized at 20×, the *sdpA* (cannibal) reporter was expressed at high levels throughout the biofilm (both the periphery and interior) based on fluorescence intensity (Fig. 1B). The *tapA* (biofilm) reporter was expressed at high levels in the periphery of the colony but at low levels in the interior (Fig. 1B). Conversely, the *dhbA* (bacillibactin) reporter was expressed more strongly in the middle layer of cells in the interior of the biofilm and very little at the periphery. This was similar to the *hag* (motility) reporter, although *hag* exhibited some narrow regions of high expression in portions of the peripheral biofilm (Fig. 1B). The range of expression patterns we observed in these data support our hypothesis that many genes are heterogeneously but reproducibly expressed in particular regions of B. subtilis biofilms. The reproducibility of the gene expression patterns observed within these completely independent colonies gave us confidence that the MSI data obtained from thin sections from analogous but different colonies would similarly be highly replicable.

### Pairing mass spectrometry and fluorescence confocal microscopy in thin-sectioned B. subtilis biofilms.

**FIG 2:**
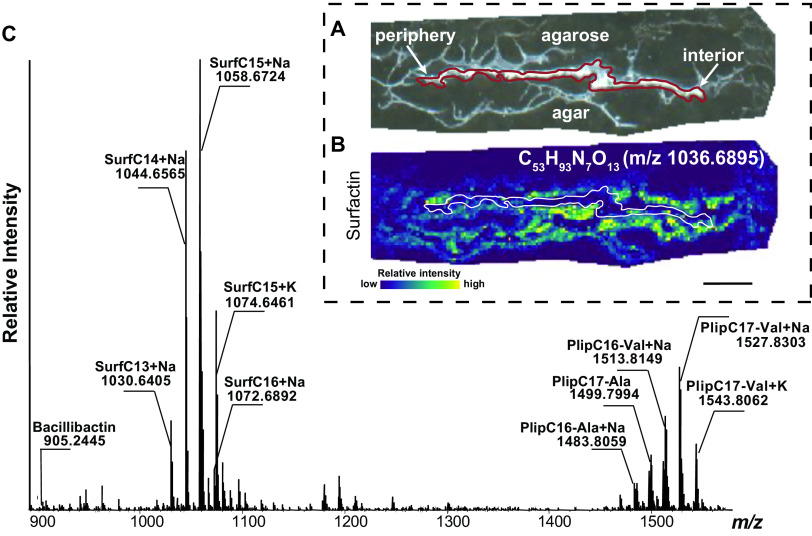
Correlative MALDI-MSI and optical microscopy of B. subtilis thin-sectioned biofilms. (A) Bright-field image of thin-sectioned B. subtilis colony grown on agar and embedded in 4% agarose with the outline of the colony in red. (B) Example of a high mass resolution ion image using MALDI-FTICR-MS to determine ion localization with the colony outlined in white. Bar, 1 mm (C) Mass spectrum of B. subtilis thin sections from ∼900 to 1500 *m/z*, with key putative molecules annotated.

We next wanted to correlate metabolite localizations with the expression patterns of these phenotypic genes of interest to visualize how the spatial metabolite network linked to phenotypic reporter expression. We grew B. subtilis biofilms on MSgg for 48 h, embedded the colonies in agarose, and thin-sectioned embedded biofilms for high mass resolution and mass accuracy MALDI-Fourier transform ion cyclotron resonance (FTICR)-MSI. The colonies used for fluorescence microscopy needed to be vapor fixed to avoid transcriptional changes during processing; however, colony fixation interferes with molecular detection by MSI (45). We therefore used equivalent but distinct replicate colonies for MSI and confocal fluorescence microscopy. Given the high replicability we observed of the biological fluorescent expression patterns occurring within B. subtilis biofilms, we had confidence that similarly consistent metabolite distributions would be observed across B. subtilis biofilms. Under bright-field microscopy conditions, we could discern three major areas of the resulting thin section prepared for MSI (Fig. 2A; see Fig. S1A in the supplemental material): (i) the colony (outlined in red/white), (ii) the agar growth medium below the colony, and (iii) the agarose embedding media on top. We could visually delineate the colony between the more fibrous agarose on top and the agar below the colony. We used MSI to spatially resolve and identify molecules with high mass resolution in these B. subtilis thin sections (Fig. 2B; Fig. S1B).

10.1128/mSystems.01038-21.1 FIG S1 Replicate B. subtilis colonies for MALDI-FTICR-MS. (A) Bright-field images of colony outlined in red. (B) High mass resolution ion images of surfactin (*m*/*z* 1036.6895); colony outlined in white. Bar, 1 mm. Download FIG S1, PDF file, 0.8 MB. Copyright © 2021 Yannarell et al. 2021 Yannarell et al. https://creativecommons.org/licenses/by/4.0/ This content is distributed under the terms of the Creative Commons Attribution 4.0 International license.

Numerous molecules colocalized with the colony, including surfactin, which also colocalized with the agar growth medium below the colony (Fig. 2B; Fig. S1B). We expected surfactin to colocalize with cells as well as the agar because of its role as both a secreted chemical cue and surfactant (30, 46, 47). Notably, there was minimal detection of surfactin in the upper embedding agarose, highlighting the robustness of our embedding methodology. Surfactin was one of the most highly detected metabolites in thin sections of B. subtilis by MSI (Fig. 2C). These surfactin data in the sagittal plane of the biofilm complement published MSI data from the top of the colony that indicates surfactin is secreted from B. subtilis 3610 colonies (30). The localization of the entire surfactin repertoire, together with accompanied molecules with molecular masses higher than 800 Da, can be explored in METASPACE (https://metaspace2020.eu/project/velickovic-2021) by browsing the bsubtilis_pos_highmz data set using the ChEBI database. At the cellular level, surfactin induces P*_tapA_* expression in neighboring cells (43); at the colony level, cells at the periphery of the colony were strongly expressing the biofilm reporter (P*_tapA_-YPet*) (Fig. 1B). However, surfactin was detected throughout the colony and not just at the colony periphery. This suggests that while surfactin may direct biofilm (i.e., *tapA*) cell differentiation at the outer edge of the colony, other factors must also be required that restrict *tapA* expression to this region rather than everywhere that surfactin is present. In addition, it raises the possibility that surfactin may play other uncharacterized roles within the interior of the biofilm. Cells producing the machinery to assemble surfactin (expressing P*_srfAA_-YPet*) were not visible by microscopy, suggesting either that the machinery was preassembled or that only a small subset of cells express the biosynthesis genes. Regardless, the MSI experiments validated the localization and secretion of surfactant from B. subtilis 3610 colony biofilms (Fig. 2B).

**FIG 3:**
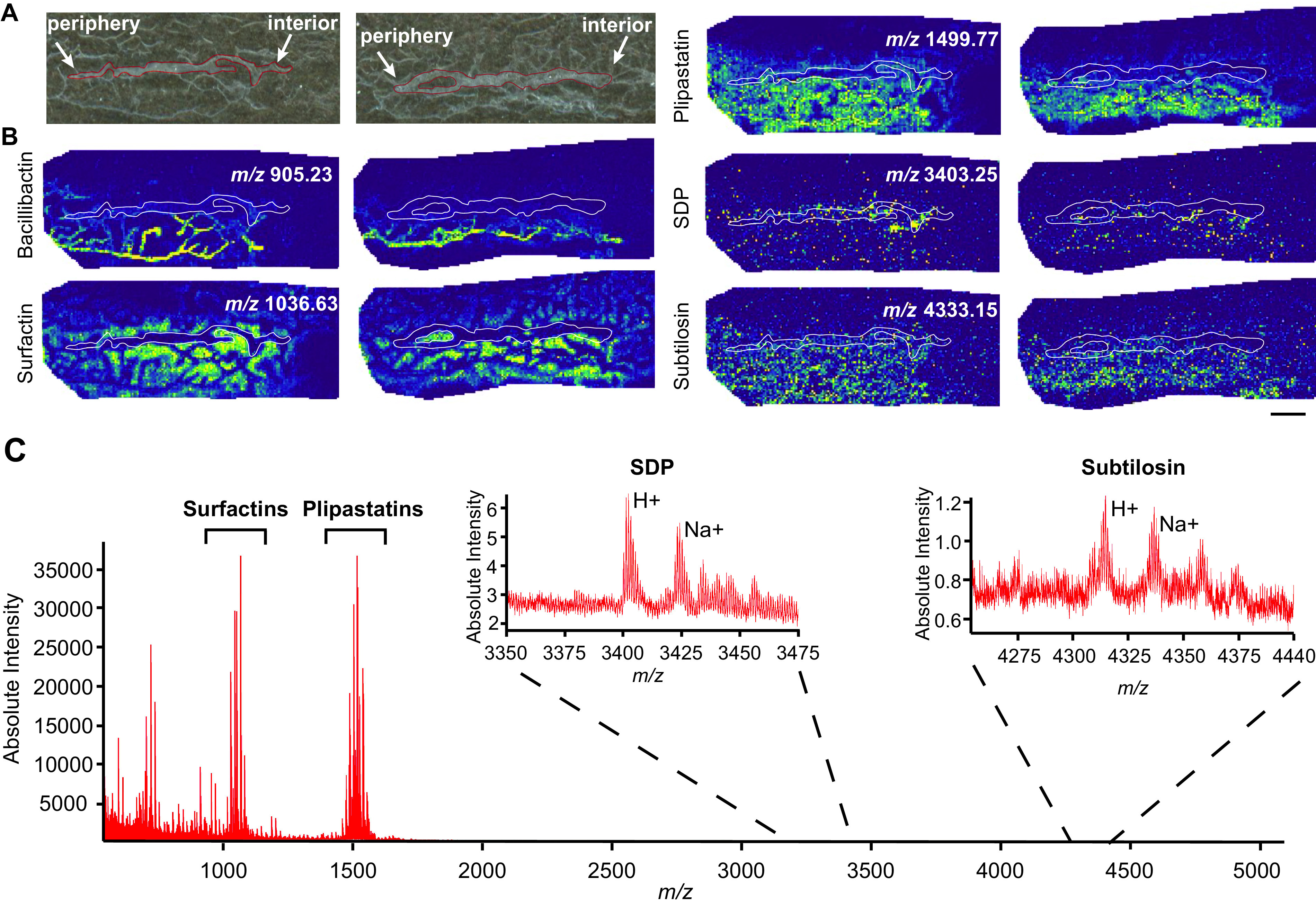
MSI of B. subtilis metabolites in thin-sectioned biofilms using MALDI-Q-TOF MS. (A) Bright-field image of replicate thin-sectioned B. subtilis colony grown on agar and embedded in agarose with the outline of the colony in red. (B) Ion distributions of bacillibactin (*m/z* 905.21), surfactin (*m/z* 1044.64), plipastatin (*m/z* 1499.77), SDP (*m/z* 3403.25), and subtilosin (*m/z* 4333.15) within B. subtilis replicate biofilms detected using a Waters Synapt G-2 mass spectrometer; colony outlines are shown in white. Bar, 1 mm. (C) Mass spectrum highlighting surfactin, plipastatin, SDP, and subtilosin peaks.

We also determined the localization of surfactin and four other B. subtilis specialized metabolites using a MALDI-quadrupole time of flight (Q-TOF)-mass spectrometry (MS) in the biofilm. This approached provided less mass resolution than the FTICR-MS, but it afforded us the ability to measure molecules at higher mass ranges, which was critical for detecting some of the specialized metabolites of B. subtilis (i.e., sporulating delaying protein [SDP] and subtilosin) (Fig. 3A) that we were not able to identify using FTICR-MS (Fig. 3A). Even though we do not have fragmentation data to confirm that the molecules detected at *m/z* 3422 and *m/z* 4334 are SDP and subtilosin, respectively, we are confident in these putative assignments based on the lack of other MS peaks in their vicinity (Fig. 3A) and previous reports using MALDI-TOF MSI of B. subtilis colonies (34). SDP can collapse protein motive force causing autolysis in self (i.e., genetically identical) and nonself cells to release nutrients and delay sporulation (29, 48). Subtilosin is known for its antimicrobial activity; however, any intracellular signaling activity of this metabolite remains unknown (49, 50). SDP localized to the periphery and subtilosin was present throughout the colony and agar when analyzed by MALDI-Q-TOF-MS (Fig. 3B).

Similar to what was observed in the FTICR-MSI analysis, surfactins and plipastatins were also detected at high levels via MALDI-Q-TOF-MS (Fig. 3B). The distributions of surfactin (*m/z* 1036) were similar in the MSI images from both mass spectrometry instruments, being strongly colocalized with the agar growth medium in both the interior and periphery of the colony. Plipastatin also colocalized with the agar below the colony and throughout the agar (Fig. 3B). Plipastatin is known to have antifungal activity (51, 52), but this metabolite is not yet described to have an intraspecies signaling role, nor does a B. subtilis plipastatin mutant exhibit an obvious biofilm defect (53). Nevertheless, we speculate that a molecule made in such high quantities is likely to play an important physiological role, potentially in later stages of biofilm development such as sporulation or germination, aspects of cellular differentiation not directly probed by our study. Bacillibactin was also present in the agar supporting colony growth, although even more strongly associated with the dried agar fibers (which are visible in the bright-field image as white areas outside the biofilm slice) (Fig. 3B).

Taken together, these MSI data (Fig. 2 and 3) indicate that we can readily detect several specialized metabolites and other molecules produced by B. subtilis. Future efforts to detect the remaining known B. subtilis specialized metabolites could be accomplished by altering the MS method used or by transitioning to another ionization source (e.g., postionization MALDI-2 or laser ablation electrospray ionization), or sample preparation method (e.g., different MALDI matrix, sample derivatization, etc.)

### Distinct molecular distributions as a basis for new biological hypotheses.

**FIG 4:**
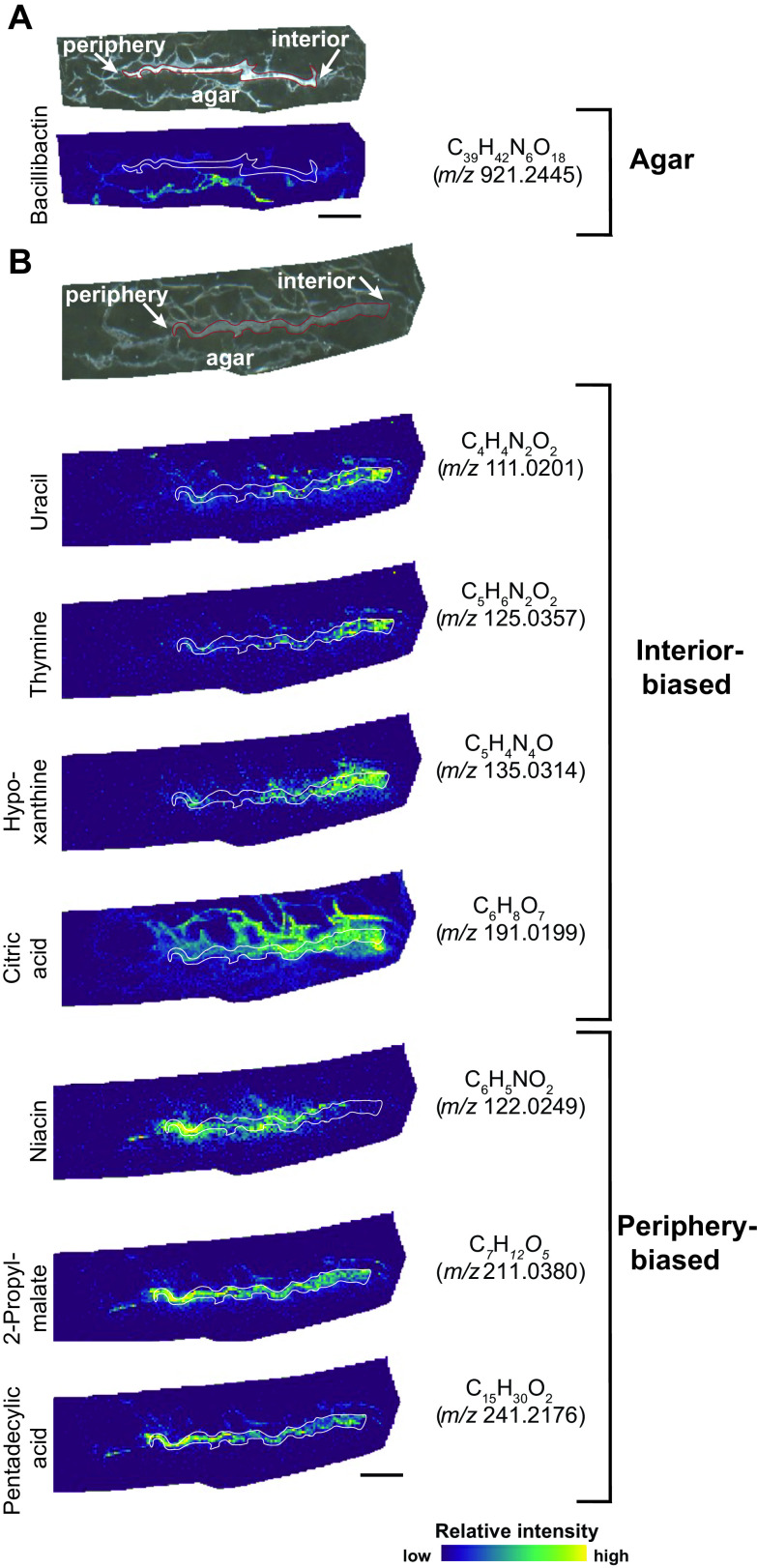
B. subtilis molecules with distinct spatial distributions. For each labeled set of images, the top images are the bright-field image of thin-sectioned B. subtilis colony grown on agar and embedded in 4% agarose, with the outline of the colony in red. Below those are the high mass resolution ion images determined by MALDI-FTICR-MS with the colony outlined in white. (A) The data set specifically focused on high masses contained an ion consistent with the siderophore bacillibactin, while data collection at lower mass ranges (B and C) contained multiple examples of molecules that colocalize with different regions of the colony. Measured *m/z* and molecular formulas within 2 ppm accuracy are shown on the right; putative molecular assignments (from METLIN) on the left. The ion images are grouped into those that are more highly detected in either the interior or periphery of the biofilm. Bars, 1 mm.

The diverse localization we observed of the phenotypic reporters in B. subtilis supported the idea that biological processes are impacted by the spatial structure of the biofilm and thus that different phenotypic processes may predominate in distinct areas of the biofilm. We not only detected some of the canonical B. subtilis specialized metabolites in our data set as noted above but also a handful of other potentially important molecules with distinct metabolic patterns across the biofilm. To understand what pathways these other metabolites in our data set may be linked to, we performed a metabolic pathway analysis using our putatively annotated molecules with the B. subtilis metabolite BSubCyc database. It is important to note that, due to mass isomerism—which is when molecules have the same number of the same kind of atoms, and therefore an identical molecular formula and molecular mass (e.g., citrate and isocitrate)—a MSI ion image has the potential to represent different molecules that it is not possible to distinguish using mass spectrometry alone. Nevertheless, we identified a number of B. subtilis pathways with high percent coverage (number of hits/total number of molecules in the pathway) based on the putative annotations from our MSI data, including: (i) alanine, aspartate, and glutamine metabolism, (ii) arginine biosynthesis, and (iii) the tricarboxylic acid (TCA) cycle (Table S2). A handful of molecules (both those that were and those that were not associated with these pathways) exhibited distinctive spatial organization within the colony (Fig. 4). One of these was the specialized metabolite bacillibactin, which we detected as being primarily localized in the agar beneath the interior of the colony (Fig. 4A). Bacillibactin is a siderophore able to scavenge iron to benefit cells within the biofilm (24, 54, 55). We predict that by 48 h cells in the colony interior/center may have exhausted their iron resources, and thus that the production of bacillibactin in the center of the colony may allow cells to access the limited amounts of iron stores still present in the agar growth medium.

10.1128/mSystems.01038-21.4 TABLE S2 MALDI-FTICR-MSI metabolic coverage of some pathways in Bacillus subtilis database based on MetaboAnalyst 5.0. Download Table S2, PDF file, 0.1 MB. Copyright © 2021 Yannarell et al. 2021 Yannarell et al. https://creativecommons.org/licenses/by/4.0/ This content is distributed under the terms of the Creative Commons Attribution 4.0 International license.

Many other detected ions similarly showed distinct spatial biases in their distribution throughout the biofilm. We show a subset of such MSI images with their putative ion assignments in Fig. 4 (replicate MSI images shown in Fig. S2). We note that the ion images for some of the smaller molecules suggest that they are diffusing away from the colony section itself into the top-embedding agarose (e.g., uracil and citric acid in Fig. 4B). We theorize that this most likely resulted during the process of overlaying the liquified agarose onto the colony before sectioning rather than as a result of MSI workflow interference. That is because we observe that the signal intensity of these metabolites corresponds tightly to the agar fibers and because the signal is absent from the areas between fibers (Fig. 4B).

10.1128/mSystems.01038-21.2 FIG S2 Replicate MSI images of ions detected by MALDI-FTICR-MS shown in Fig. 4. Download FIG S2, PDF file, 0.6 MB. Copyright © 2021 Yannarell et al. 2021 Yannarell et al. https://creativecommons.org/licenses/by/4.0/ This content is distributed under the terms of the Creative Commons Attribution 4.0 International license.

One ion image putatively labeled as citric acid, a TCA cycle intermediate, was abundant in the interior of the colony but less so in the periphery of the colony (Fig. 4B). The TCA cycle is upregulated early in biofilm development (11), suggesting these intermediates may be accumulating in the interior of the colony where “older” cells (which are no longer expressing biofilm matrix genes and likely not growing as rapidly as cells on the expanding periphery of the biofilm) are localized at 48 h. In addition, several ions putatively annotated as uracil, thymine, and hypoxanthine colocalize to the interior of the biofilm (similar to the *dhbA* and *hag* reporters), while ions that can be ascribed to niacin colocalize to the periphery of the biofilm, more aligned with the distribution observed for the *tapA* reporter (Fig. 1 and Fig. 4B). Uracil, thymine, and hypoxanthine are nucleobases in B. subtilis. We hypothesize that these molecules may accumulate in the interior of the biofilm (56) where cells are not actively replicating (57, 58). Niacin (nicotinic acid) is a precursor of the coenzymes NAD and NADP, which are essential for cellular processes (59). As far as we are aware, niacin has not been directly studied in B. subtilis biofilms, but the accumulation we observe supports data associating an increase in *tapA* biofilm matrix reporter gene expression in cells with a decrease in respiration efficiency (60). However, this relationship appears to break down at the extreme outer edge of the colony, where *tapA* expression remains high (Fig. 1) while the putative niacin ion intensity declines (Fig. 4B). Last, a few putative molecules, including 2-propylmalate and pentadecylic acid, strongly colocalize throughout the colony, with both exhibiting higher intensities near the peripheral edge of the colony (Fig. 4B). Better understanding the potential relationships between these and other metabolites and the cells within B. subtilis biofilms could be explored in future work that adds back purified compounds to test the impact of these molecules on gene expression patterns or via genetic knockouts that eliminate key metabolites or their precursors.

The entire set of spatially resolved metabolites putatively annotated from our analyses can be visualized in METASPACE (https://metaspace2020.eu/project/velickovic-2021). Browsing is possible through the bsubtilis_pos_lowmz and bsubtilis_neg_lowmz data sets using our semicustomized B. subtilis database (PAMDB and ECMDB are also available), which provides the most comprehensive views into the complex spatial metabolomic network of this bacterial biofilm. Future efforts could build on our results to maximally exploit the benefits of MSI in this multimodal work. For example, our current spatial resolution of 50 μm, although sufficient to capture different features of the biofilm, still averages signal from ∼100 bacterial cells in a specific biofilm layer. We are currently working on benchmarking postionization MALDI-2 technology (61) for microbial research. This approach should increase the sensitivity of MALDI analysis and allow us to go to submicrometric spatial resolutions, allowing us to visualize molecular heterogeneity at such finer spatial scales. In addition, introduction of orthogonal spatial measurements (28) could narrow down even further the list of tentative molecules for a given ion image (since our current METASPACE results list all natural mass isomers for a particular molecular formula).

Taken together, these data sets allow us to qualitatively correlate molecular localization (from MSI images) with gene expression patterns (based on fluorescence microscopy) at high spatial resolution within the 3D depths of bacterial biofilms. We can use this method to generate new hypotheses regarding the associations of particular putative annotated species with known bacterial gene expression pathways. These data will also stimulate ideas regarding whether previously uncharacterized molecules that exhibited nonuniform localizations may be important in either driving cellular differentiation or acting as biomarkers for other genetically distinct, spatially defined subpopulations of cells.

## MATERIALS AND METHODS

### Bacterial strains and growth conditions.

B. subtilis strains were cultured on lysogeny broth (LB)-Lennox medium (10 g/liter tryptone, 5 g/liter yeast extract, 5 g/liter NaCl, 1.5% agar) at 30°C for 16 to 18 h with antibiotics as necessary. Colony biofilms were grown on MSgg medium (5 mM potassium phosphate [pH 7], 100 mM morpholinepropanesulfonic acid [MOPS; pH 7], 2 mM MgCl_2_, 700 μM CaCl_2_, 50 μM MnCl_2_, 50 μM FeCl_3_, 1 μM ZnCl_2_, 2 μM thiamine, 0.5% glycerol, 0.5% glutamate) with 1.5% agar (30-ml plates). Antibiotics (final concentrations) were used as follows: MLS (1 μg/ml erythromycin, 25 μg/ml lincomycin) and chloramphenicol (5 μg/ml).

### Construction of B. subtilis reporter strains.

All primers, plasmids, and strains used in this study can be found in Table S1 in the supplemental material. The transcriptional reporter plasmids constructed for this study (see plasmids in Table S1) containing *YPet* were derived from pES045 (*amyE*::P*_spacC_*-*YPet*) (62). Specifically, the *spacC* promoter (P*_spacC_*) was removed by digestion with EcoRI and HindIII and replaced with gene-specific promoter sequences. The promoter sequences were amplified from B. subtilis wild-type genomic DNA (see primers in Table S1) and inserted into the base plasmid by isothermal assembly (63).

A plasmid containing the gene for mTurquoise2 (*mTurq*) was generated using primer ES395 and primer ES315 (Table S1) to amplify *mTurq* from GL-FP-31. The fragment was cloned into plasmid pDR183 [*lacA*::*mls*] (64) digested with SalI and EcoRI. To create *mTurq* reporters, we amplified promoter sequences from B. subtilis wild-type genomic DNA (see primers in Table S1), digested with NheI and SalI, and inserted into the pDR183-*mTurq* base plasmid (pES069) using isothermal assembly.

Upon final construction, the linearized plasmids were transformed into B. subtilis 168 cells grown to stationary phase. Cells containing *YPet* reporters were plated on LB-Lennox-chloramphenicol to select for transformants. Cells containing *mTurq* reporters were plated on LB-Lennox-MLS to select for transformants. Phage transduction was carried out as previously described (65). B. subtilis *mTurq* reporters were used as the donor strains and grown to 37°C in TY broth until the culture reached an optical density at 600 nm (OD_600_) of 1.0. Cells were infected with SPP1 phage stock and plated on 0.5% TY soft top agar, overlaid on TY 1.5% agar plates, and incubated at 37°C for 8 to 16 h. B. subtilis donor phage plaques were collected and pelleted using a clinical centrifuge. Three hundred microliters of supernatant was used to infect B. subtilis 3610 wild-type and B. subtilis *YPet* reporter strains (recipient cells) to construct single and dual-fluorescent reporters, respectively. The cells were then plated on LB-Lennox with 10 mM citrate and MLS (to which the donor *mTurq* reporter strains were resistant). Plates were incubated at 37°C for 12 to 24 h. Three colonies were picked from each phage transduction and struck on LB-Lennox plates with MLS and citrate to select for B. subtilis cells that contained *mTurq* reporters. For strains containing dual-fluorescent reporters, strains were then restruck on LB-Lennox-chloramphenicol to select for strains containing both *mTurq* and *YPet* reporters. Cells were spotted on MSgg and incubated at 30°C to ensure growth; growth on this recipe of MSgg indicates the cells have a 3610 background rather than a 168 background (which is a triple amino acid auxotroph). Colony morphology of reporter strains were also compared to that of the wild type, as morphology should be identical.

### Thin sectioning.

The thin sectioning protocol was adapted from the protocols of Vlamakis and colleagues (20) and Marlow and colleagues (66). For both MSI and microscopy, B. subtilis strains were cultured on MSgg as described above. For colonies used in MSI, biofilm-agar blocks were quartered, transferred to a 15 mm × 15 mm × 5 mm mold (catalog no. 22-363-553; Fisher), and snap-frozen at −80°C. The colony was then overlaid with 4% (wt/vol) agarose (catalog no. 50181; Lonza) and frozen at −80°C for 15 min. The blocks were then transferred to −20°C for 30 min to equilibrate. Colony blocks were mounted to the chuck with distilled deionized H_2_O and sliced to 20-μm-thick cross sections using a cryomicrotome (Thermo Cryostar NX70). For colonies used in microscopy, colonies were vapor fixed with 8% paraformaldehyde before their removal from the plate for embedding and cryosectioning as described above (67).

### MALDI-FTICR-MSI sample preparation, data acquisition, and data processing.

Thin sections were mounted on indium tin oxide (ITO)-coated glass slides (catalog no. 8237001; Bruker) and stored at −80°C until analysis. Frozen slides were transferred to the lyophilization chamber and freeze-dried for 45 min. MALDI matrix application was performed using a TM-Sprayer (M3 model; HTX Technologies). For analysis in positive ionization mode, 40 mg/ml of 2,5-dihydroxybenzoic acid (DHB) in 70% methanol (MeOH) was sprayed with 16 passes at 50 μl/min, 70°C, a spray spacing of 3 mm, and a spray velocity of 1200 mm/min. For analysis in negative ionization mode, 7 mg/ml of *N*-(1-naphthyl) ethylenediamine dihydrochloride (NEDC) in 70% MeOH was sprayed with 8 passes at 1,200 μl/min, 75°C, a spray spacing of 3 mm, and a spray velocity of 1200 mm/min. MSI was performed on a 15-Tesla MALDI-FTICR-MS (Bruker Daltonics) equipped with SmartBeam II laser source (355 nm, 2 kHz) using 200 shots/pixel with a frequency of 2 kHz and a 50-μm step size. For small-molecule analysis, FTICR-MS was operated to collect *m/z* 92 to 700, using a 209-ms transient, which translated to a mass resolution *R* of ∼60,000 at 400 *m/z*. For analysis of plipastatins and surfactins, FTICR-MS was operated to collect *m/z* 800 to 2,500, using a 908-ms transient, which translated to a mass resolution *R* of ∼260,000 at 400 *m/z*. Data were acquired using FlexImaging (v 4.1; Bruker Daltonics), and image processing, segmentation, colocalization analysis, and visualization were performed using SCiLS (Bruker Daltonics). The list of *m/z* values that colocalize with the colony were uploaded to the METLIN (https://metlin.scripps.edu) and BSubCyc database (https://bsubcyc.org) for putative molecular annotations based only on accurate *m/z*, which was secured by using a 2-ppm window during the search. Putatively annotated compounds were then uploaded to the Pathway Analysis module of MetaboAnalyst 5.0 (https://www.metaboanalyst.ca/) with search restricted to the B. subtilis database for insights into metabolic pathway impact. imzML files (created by SCiLS) of our analyses were also uploaded to METASPACE (68) for metabolite annotation based not only on accurate *m/z* but also on isotopologue spatial match and spatial metabolite knowledgebase. For this purpose, we created a METASPACE-compatible B. subtilis database using compounds present in BSubCyc database collection (https://bsubcyc.org). METASPACE annotations can be browsed at https://metaspace2020.eu/project/velickovic-2021. Note that METASPACE uses by default 3-ppm window in its annotation engine.

### MALDI-Q-TOF (Synapt) MSI data acquisition and data processing.

Cryosections of B. subtilis colonies were prepared as described above for MALDI-FTICR-MSI but analyzed using a Synapt G2-Si (Waters) powered by a solid-state laser with a repetition rate of 2.5 KHz for assessing spatial distribution of larger oligopeptides (>3,000 *m/z*) within the colony. The instrument was operated in sensitivity mode and 32,000 (32K) quadrupole collecting positive ions in a *m/z* range of 500 to 5,000 with 50-μm step size. Imaging data were processed using HDI V1.5 software, converted to imzML format, and uploaded to the SCiLS software for visualization of mass spectra and ion images.

### Optical microscopy.

Fixed thin sections for microscopy were attached to VWR Superfrost Plus slides (catalog no. 48311-703) and stored at −20°C. Before imaging, slides were placed at room temperature, overlaid with mounting medium ProLong Gold Antifade Mountant (catalog no. P10144; ThermoFisher), and a 25 × 25 mm coverslip (catalog no. 12-548-C; Fisher). Sections were imaged using a Zeiss 710 laser scanning confocal microscope equipped with a 20× EC Plan Neofluar objective.

### Data availability.

We created a METASPACE-compatible B. subtilis database using compounds present in BSubCyc database collection (https://bsubcyc.org). METASPACE annotations can be browsed at https://metaspace2020.eu/project/velickovic-2021.
