# Supplementary material for: Direct Visualization of Chemical Cues and Cellular Phenotypes throughout Bacillus subtilis Biofilms
Source: mSystems. 2021 Nov 23;6(6):e01038-21. doi: 10.1128/mSystems.01038-21 (PMC8609973; doi:10.1128/mSystems.01038-21)
Supplement: TABLE S1 [file msystems.01038-21-st001.pdf]

Table S1. Primers, Plasmids, and Strains used in this study

Primers used in this study

Primer Name	Description	Sequence (5' to 3')
ES735	P_{hag} insertion in *lacA::mTurq* (pES069) to construct pES074	gatcctagcatgcatgctagcgaattgacgccccaaagc
ES736	P_{hag} insertion in *lacA::mTurq* (pES069) to construct pES074	tagttcctccttatgtgtcgacctgaatatgttgttaaggcacg
ES753	P_{pksC} insertion in *lacA::mTurq* (pES069) to construct pES084	gatcctagcatgcatgctagcatccttctgaatctattatcc
ES754	P_{pksC} insertion in *lacA::mTurq* (pES069) to construct pES084	tagttcctccttatgtgtcgactccgattagtagatgtgtttc
ES777	P_{tapA} insertion in *amyE::Ypet* (pES045) to construct pES099	aagctgtcaaacatgagaattcctcagagttaaatggtattgc
ES778	P_{tapA} insertion in *amyE::Ypet* (pES045) to construct pES112	gtagttcctccttatgtaagcttttgtaaaacactgtaacttgatatg
ES779	P_{bacA} insertion in *amyE::Ypet* (pES045) to construct pES100	aagctgtcaaacatgagaattcaacaaagtttctaaattcctataattaaaaac
ES780	P_{bacA} insertion in *amyE::Ypet* (pES045) to construct pES100	gtagttcctccttatgtaagcttatcttttaaaattttaagtaaattttatccag
ES785	P_{dhbA} insertion in *amyE::Ypet* (pES045) to construct pES103	aagctgtcaaacatgagaattcagacatactcagccttgc
ES786	P_{dhbA} insertion in *amyE::Ypet* (pES045) to construct pES103	gtagttcctccttatgtaagctttcgctctaaatgccaaac
ES787	P_{hag} insertion in *amyE::Ypet* (pES045) to construct pES104	aagctgtcaaacatgagaattcgaattgacgccccaaagc
ES788	P_{hag} insertion in *amyE::Ypet* (pES045) to construct pES104	gtagttcctccttatgtaagcttctgaatatgttgttaaggcacg
ES795	P_{sdpA} insertion in *amyE::Ypet* (pES045) to construct pES108	aagctgtcaaacatgagaattcttcaattctgggaactttttaag
ES796	P_{sdpA} insertion in *amyE::Ypet* (pES045) to construct pES108	gtagttcctccttatgtaagcttttgatgtagattacctcctc
ES825	P_{skfA} insertion in *lacA::mTurq* (pES069) to construct pES114	gatcctagcatgcatgctagcacttcattcatttaaccaaag
ES826	P_{skfA} insertion in lacA::mTurq (pES069) to construct pES114	tagttcctccttatgtgtcgaccaatttttgcatagagtctattg
ES879	P_{csn} insertion in *lacA::mTurq* (pES069) to construct pES116	gatcctagcatgcatgctagctttctgccagctccttattttttatg
ES880	P_{csn} insertion in lacA::mTurq (pES069) to construct pES116	tagttcctccttatgtgtcgactctaaatattgtaaatacagactcagtatac
ES997	RBS and mTurq insertion in *lacA::erm* (pDR183) to construct pES069	gctagcatctcgagtagtcgacacataaggaggaactactatggtatcaaaaggggaagagttg
ES998	RBS and mTurq insertion in *lacA::erm* (pDR183) to construct pES069	cccggggagctcatgaattctttgcctggcggcagtag
ES999	P_{aprE} insertion in *lacA::mTurq* (pES069) to construct pES119	gtaggatcctagcatgcatgctagctctatccctttctgtaaag
ES1000	P_{aprE} insertion in *lacA::mTurq* (pES069) to construct pES119	tagtagttcctccttatgtgtcgacaaaaattcagagtagacttacttaaaag

Plasmids used in this study

Plasmid	Genotype	Reference
pDR183	*lacA::erm (amp)*	Doan et al 2005
GL-FP-31	m*Turquiose2*	Garner lab collection
pES045	*amyE*::P_{spacC}-*Ypet (cam)*	Yannarell et al. 2019
pES069	*lacA*::m*Turq (erm)*	This study
pES074	*lacA*::P_{hag}-m*Turq (erm)*	This study
pES084	*lacA*::P_{pksC}-m*Turq (erm)*	This study
pES099	*amyE*::P_{tapA}-*Ypet (cam)*	This study
pES100	amyE::P_{bacA}-*Ypet (cam)*	This study
pES103	*amyE*::P_{dhbA}-*Ypet (cam)*	This study
pES104	*amyE*::P_{hag}-*Ypet (cam)*	This study
pES108	*amyE*::P_{sdpA}-*Ypet* (cam)	This study
pES114	*lacA*::P_{skfA}-m*Turq (erm)*	This study
pES116	*lacA*::P_{csn}-m*Turq (erm)*	This study
pES119	*lacA*::P_{aprE}-m*Turq (erm)*	This study

Strains used in this study

ES Strain (Pseudonym)	Bacterial species/ strain	Genotype	Source (Reference)
NCIB3610	*Bacillus subtilis* 3610	Wild type	Shank lab collection
2056	*Bacillus subtilis* 3610	*lacA*::P_{pksC}-*mTurq (erm)*	This study
2057	*Bacillus subtilis* 3610	*lacA*::P_{skfA}-*mTurq (erm)*	This study
2059	*Bacillus subtilis* 3610	*lacA*::P_{hag}-*mTurq (erm)*	This study
2060	*Bacillus subtilis* 3610	*lacA*::P_{aprE}-*mTurq (erm)*	This study
2060	*Bacillus subtilis* 3610	*lacA*::P_{csn}-*mTurq (erm)*	This study
2089	*Bacillus subtilis* 168	*amyE*::P_{hag}-*YPet (cam)*	This study
2090	*Bacillus subtilis* 168	*amyE*::P_{tapA}-*YPet (cam)*	This study
2091	*Bacillus subtilis* 168	*amyE*::P_{sdpA}-*YPet (cam)*	This study
2096	*Bacillus subtilis* 168	*amyE*::P_{dhbA}-*YPet (cam)*	This study
2097	*Bacillus subtilis* 168	*amyE*::P_{bacA}-*YPet (cam)*	This study
2106	*Bacillus subtilis* 3610	*amyE*::P_{hag}-*YPet* (cam)	This study
2107	*Bacillus subtilis* 3610	*amyE*::P_{tapA}-*YPet (cam)*	This study
2108	*Bacillus subtilis* 3610	*amyE*::P_{sdpA}-*YPet (cam)*	This study
2113	*Bacillus subtilis* 3610	*amyE*::P_{dhbA}-*YPet (cam)*	This study
2114	*Bacillus subtilis* 3610	*amyE*::P_{bacA}-*YPet (cam)*	This study
2174	*Bacillus subtilis* 3610	*lacA*::P_{bacA}-*mTurq (erm)*	This study
2184	*Bacillus subtilis* 3610	*amyE*::P_{tapA}-*YPet*; *lacA*::P_{hag}-*mTurq*	This study
2191	*Bacillus subtilis* 3610	*amyE*::P_{tapA}-*YPet*; *lacA*::P_{bacA}-*mTurq*	This study
2223	*Bacillus subtilis* 3610	*amyE*::P_{hag}-*YPet*; *lacA*::P_{pksC}-*mTurq*	This study
2224	*Bacillus subtilis* 3610	*amyE*::P_{hag}-*YPet*; *lacA*::P_{bacA}-*mTurq*	This study
2248	*Bacillus subtilis* 3610	*amyE*::P_{sdpA}-*YPet*; *lacA*::P_{skfA}-*mTurq*	This study
2249	*Bacillus subtilis* 3610	*amyE*::P_{sdpA}-*YPet*; *lacA*::P_{aprE}-*mTurq*	This study
2279	*Bacillus subtilis* 3610	*amyE*::P_{dhbA}-*YPet*; *lacA*::P_{bacA}-*mTurq*	This study
2286	*Bacillus subtilis* 3610	*amyE*::P_{dhbA}-*YPet*; *lacA*::P_{csn}- *mTurq*	This study
2289	*Bacillus subtilis* 3610	*amyE*::P_{dhbA}-*YPet*; *lacA*::P_{aprE}-*mTurq*	This study
2299	*Bacillus subtilis* 3610	*amyE*::P_{bacA}-*YPet*; *lacA*::P_{skfA}-*mTurq*	This study
2230	*Bacillus subtilis* 3610	*amyE*::P_{bacA}-*YPet*; *lacA*::P_{aprE}-*mTurq*	This study
2509	*Bacillus subtilis* 168	*lacA*::P_{pksC}-*mTurq (erm)*	This study
2510	*Bacillus subtilis* 168	*lacA*::P_{skfA}-*mTurq (erm)*	This study
2511	*Bacillus subtilis* 168	*lacA*::P_{hag}-*mTurq (erm)*	This study
2512	*Bacillus subtilis* 168	*lacA*::P_{aprE}-*mTurq (erm)*	This study
2522	*Bacillus subtilis* 168	*lacA*::P_{bacA}-*mTurq (erm)*	This study
