# Supplementary figures and images for: Direct Visualization of Chemical Cues and Cellular Phenotypes throughout Bacillus subtilis Biofilms
Source: mSystems. 2021 Nov 23;6(6):e01038-21. doi: 10.1128/mSystems.01038-21 (PMC8609973; doi:10.1128/mSystems.01038-21)

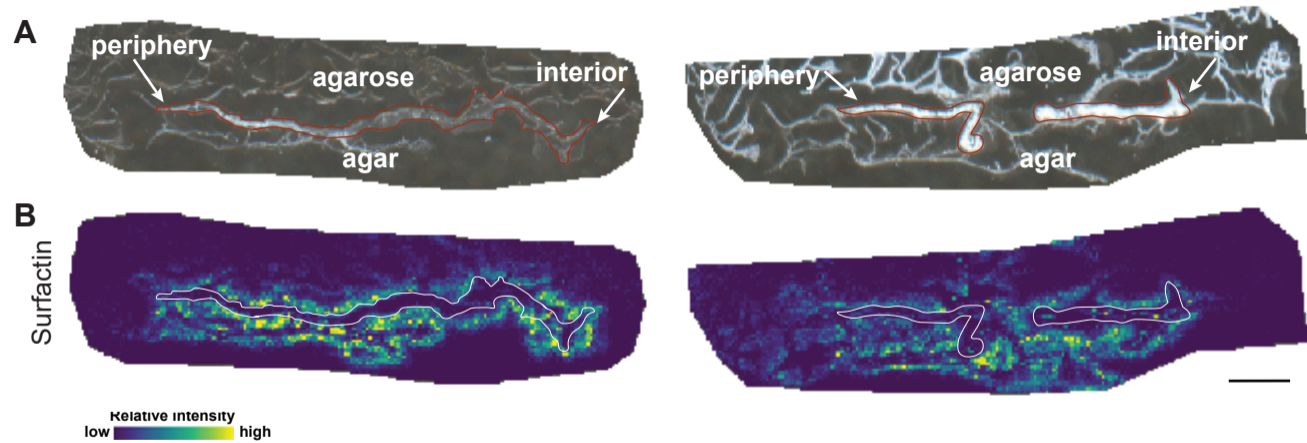

Figure S1.

Supplement: FIG S1 [file msystems.01038-21-sf001.pdf]

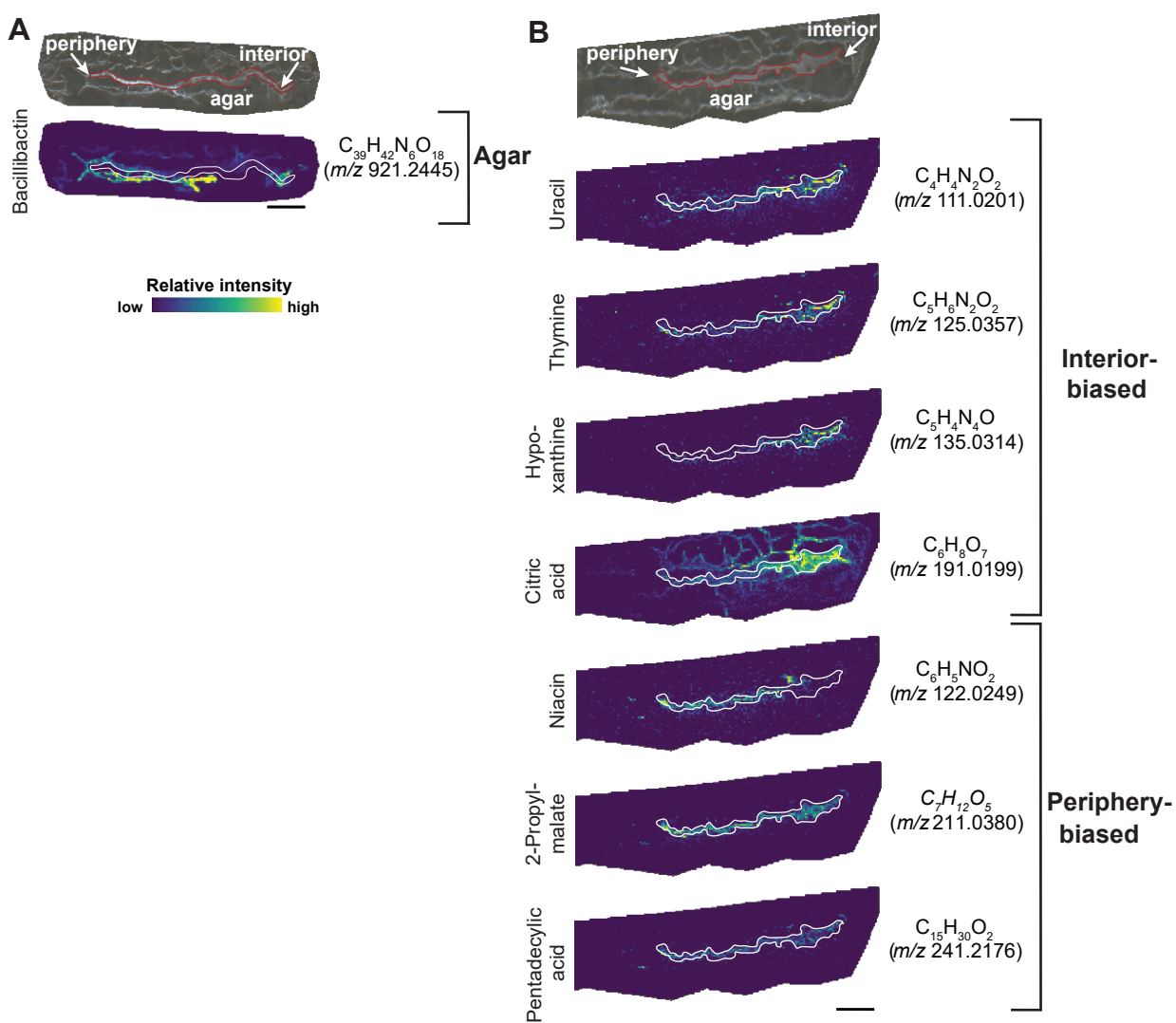

Figure S2.

Supplement: FIG S2 [file msystems.01038-21-sf002.pdf]
