# Supplementary material for: Direct Visualization of Chemical Cues and Cellular Phenotypes throughout Bacillus subtilis Biofilms
Source: mSystems. 2021 Nov 23;6(6):e01038-21. doi: 10.1128/mSystems.01038-21 (PMC8609973; doi:10.1128/mSystems.01038-21)
Supplement: TABLE S2 [file msystems.01038-21-st002.pdf]

Table S2. MALDI-FTICR-MSI metabolic coverage of some pathways in *Bacillus subtilis* database based on MetaboAnalyst 5.0.

Pathway	Kegg	Compound	% coverage
Alanine, aspartate and glutamate metabolism			21.7
	C00049	L-Aspartate	
	C00025	L-Glutamate	
	C00334	4-Aminobutanoate	
	C00064	L-Glutamine	
	C00042	Succinate	
Arginine biosynthesis			22.2
	C00062	L-Arginine	
	C00025	L-Glutamate	
	C00049	L-Aspartate	
	C00064	L-Glutamine	
Citrate cycle (TCA cycle)			20.0
	C00068	Thiamin diphosphate	
	C00042	Succinate	
	C00311	Isocitrate	
	C00158	Citrate	
Butanoate metabolism			19.0
	C00334	4-Aminobutanoate	
	C00497	(R)-Malate	
	C00042	Succinate	
	C06010	(S)-2-Acetolactate	
Biosynthesis of siderophore group nonribosomal peptides			40.0
	C00251	Chorismate	
	C00885	Isochorismate	
Arginine and proline metabolism			16.7
	C00062	L-Arginine	
	C00025	L-Glutamate	
	C00334	4-Aminobutanoate	
	C00315	Spermidine	
Nicotinate and nicotinamide metabolism			20.0
	C00049	L-Aspartate;	
	C00253	Nicotinate	
	C00042	Succinate	
Nitrogen metabolism			28.6
	C00064	L-Glutamine	
	C00025	L-Glutamate	
D-Glutamine and D-glutamate metabolism			28.6
	C00064	L-Glutamine	
	C00025	L-Glutamate	
Glutathione metabolism			16.7
	C01879	5-Oxoproline	
	C00025	L-Glutamate	
	C00315	Spermidine	
Glycolysis / Gluconeogenesis			13.3
	C00068	Thiamin diphosphate	
	C00103	D-Glucose 1-phosphate	
	C01172	beta-Glucose 6-phosphate	
Glyoxylate and dicarboxylate metabolism			11.8
	C00158	Citrate	
	C00025	L-Glutamate	
	C00311	Isocitrate	
	C00064	L-Glutamine	
Ubiquinone and other terpenoid-quinone biosynthesis			18.2
	C00885	Isochorismate	
	C00251	Chorismate	
Carbapenem biosynthesis			33.3
	C00025	L-Glutamate	
Novobiocin biosynthesis			33.3
	C00254	Prephenate	

Acarbose and validamycin biosynthesis			33.3
	C00103	D-Glucose 1-phosphate	
Sulfur metabolism			13.3
	C00979	O-Acetyl-L-serine	
	C00042	Succinate	
Cysteine and methionine metabolism			9.3
	C02989	L-Methionine S-oxide	
	C00979	O-Acetyl-L-serine	
	C00049	L-Aspartate	
	C02356	(S)-2-Aminobutanoate	
Polyketide sugar unit biosynthesis			20.0
	C00103	D-Glucose 1-phosphate	
beta-Alanine metabolism			16.7
	C00049	L-Aspartate	
Fructose and mannose metabolism			10.0
	C00275	D-Mannose 6-phosphate	
Pantothenate and CoA biosynthesis			9.5
	C06010	(S)-2-Acetolactate	
	C00049	L-Aspartate;	
Glycerophospholipid metabolism			9.5
	C00093	sn-Glycerol 3-phosphate	
	C00623	sn-Glycerol 1-phosphate	
Phenylalanine, tyrosine and tryptophan biosynthesis			9.1
	C00254	Prephenate	
	C00251	Chorismate	
Monobactam biosynthesis			12.5
	C00049	L-Aspartate	
Cyanoamino acid metabolism			12.5
	C00049	L-Aspartate	
Tyrosine metabolism			11.1
	C00042	Succinate	
C5-Branched dibasic acid metabolism			11.1
	C06010	(S)-2-Acetolactate	
Pentose phosphate pathway			7.7
	C01172	beta-D-Glucose 6-phosphate	
Streptomycin biosynthesis			10.0
	C00103	D-Glucose 1-phosphate	
Ascorbate and aldarate metabolism			10.0
	C00679	5-Dehydro-4-deoxy-D-glucarate	
Propanoate metabolism			7.4
	C00042	Succinate	
	C00068	Thiamin diphosphate	
Aminoacyl-tRNA biosynthesis			6.7
	C00062	L-Arginine	
	C00049	L-Aspartate	
	C00025	L-Glutamate	
Taurine and hypotaurine metabolism			9.1
	C00025	L-Glutamate	
Vitamin B6 metabolism			9.1
	C00018	Pyridoxal phosphate	
Riboflavin metabolism			8.3
	C00016	FAD	
Valine, leucine and isoleucine degradation			6.7
	C00068	Thiamin diphosphate	
	C03284	L-3-Aminoisobutanoate	
Purine metabolism			6.0
	C00064	L-Glutamine	
	C00147	Adenine	
	C00242	Guanine	
	C00262	Hypoxanthine	
Biosynthesis of secondary metabolites - other antibiotics			7.1

	C00254	Prephenate	
Lysine biosynthesis			6.7
	C00049	L-Aspartate;	
Pyrimidine metabolism			5.7
	C00064	L-Glutamine	
	C00106	Uracil	
Histidine metabolism			5.6
	C00025	L-Glutamate	
Inositol phosphate metabolism			5.6
	C01177	Inositol 1-phosphate	
